# Robust Spike-Based Continual Meta-Learning Improved by Restricted Minimum Error Entropy Criterion

**DOI:** 10.3390/e24040455

**Published:** 2022-03-25

**Authors:** Shuangming Yang, Jiangtong Tan, Badong Chen

**Affiliations:** 1 School of Electrical and Information Engineering, Tianjin University, Tianjin 300072, China; yangshuangming@tju.edu.cn (S.Y.); shuangmingyang@tju.edu.cn (J.T.); 2 Institute of Artificial Intelligence and Robotics, Xi’an Jiaotong University, Xi’an 710049, China

**Keywords:** spiking neural network, meta-learning, information theoretic learning, minimum error entropy, artificial general intelligence

## Abstract

The spiking neural network (SNN) is regarded as a promising candidate to deal with the great challenges presented by current machine learning techniques, including the high energy consumption induced by deep neural networks. However, there is still a great gap between SNNs and the online meta-learning performance of artificial neural networks. Importantly, existing spike-based online meta-learning models do not target the robust learning based on spatio-temporal dynamics and superior machine learning theory. In this invited article, we propose a novel spike-based framework with minimum error entropy, called MeMEE, using the entropy theory to establish the gradient-based online meta-learning scheme in a recurrent SNN architecture. We examine the performance based on various types of tasks, including autonomous navigation and the working memory test. The experimental results show that the proposed MeMEE model can effectively improve the accuracy and the robustness of the spike-based meta-learning performance. More importantly, the proposed MeMEE model emphasizes the application of the modern information theoretic learning approach on the state-of-the-art spike-based learning algorithms. Therefore, in this invited paper, we provide new perspectives for further integration of advanced information theory in machine learning to improve the learning performance of SNNs, which could be of great merit to applied developments with spike-based neuromorphic systems.

## 1. Introduction

In recent years, deep learning has shown a superior performance that exceeds the human-level performance in various types of individual narrow tasks [1]. However, in comparison with human intelligence that can learn to learn continually in order to execute unlimited tasks, the current successful deep learning methods still have a lot of drawbacks and limitations. In fact, humans can learn to learn by accumulating knowledge across their life time, which is a great challenge for artificial neural networks (ANNs) [2]. From this point of view, continual meta-learning aims at realizing machine intelligence at a higher level by providing machines with the meta-learning capability of learning to learn continually [3].

The human brain can realize meta-learning continually and avoid the catastrophic forgetting problem based on a combination of neural mechanisms [4]. The catastrophic forgetting problem is the critical challenge for developing the capability of continual meta-learning [5]. The human brain has implemented an efficient and scalable mechanism for continual learning based on neuronal activity patterns that represent previous experiences [6]. Neurons communicate with each other and process the neural information by using neural spikes, which is one of the most critical fundamental mechanism in the brain. Based on this mechanism, the human brain can realize superior performance in different aspects, such as low power consumption and high spatio-temporal processing capability [7]. Therefore, implementing a brain-inspired continual meta-learning algorithm based on spike patterns and the brain’s mechanisms is a promising technique.

The spiking neural network (SNN) uses the biologically plausible neuron model based on spiking dynamics, while the conventional ANN only uses the neurons based on a static rate [8]. SNNs are applied to reproduce the brain’s mechanisms and to deal with the cognitive tasks [9]. In addition, the neuromorphic hardware based on SNNs can realize high performance in artificial intelligence tasks, including low power consumption, high noise tolerance, and low computation latency [10]. Previous neuromorphic hardware researches have proven these advantages by using various types of tasks, such as Tianjic, Loihi, BiCoSS, CerebelluMorphic, and LaCSNN [11,12,13,14,15]. Researchers have proposed SNN models to realize the short-term memory capability in a spike-based framework [16]. However, the current SNN models still suffer from the continual meta-learning problem under the non-Gaussian noise, and no previous study has solved this problem. Therefore, this is the focus of this study.

Information theoretic learning (ITL) has attracted increasing attention in the field of machine learning in recent years to improve the learning robustness and enhance the explainable capability [17,18,19]. Previously, Chen et al. proposed researches focusing on maximum correntropy theory and minimum error entropy criteria to improve the robustness of machine learning theory [20,21,22]. In addition, a series of entropy-based learning algorithms have been presented to deal with the robustness improvement of machine learning models, including guided complement entropy and fuzzy entropy [23,24,25]. Nevertheless, there is no application of the ITL-based approach in the spike-based continual meta-learning to improve its learning robustness. Therefore, in this invited article, we aim to propose a novel approach to deal with this challenging problem. A novel model is presented, which is called meta-learning with minimum error entropy (MeMEE). We test the meta-learning capability of the proposed SNN model. Then, we investigate the robust working memory capability in non-Gaussian noise. Finally, the robust transfer learning performance is explored under a non-Gaussian noisy condition. Experimental results strongly suggest the robust meta-learning capability of the SNN model with a working memory feature in a non-Gaussian noisy environment.

## 2. Materials and Methods

### 2.1. SNN Model

**Figure 1:**
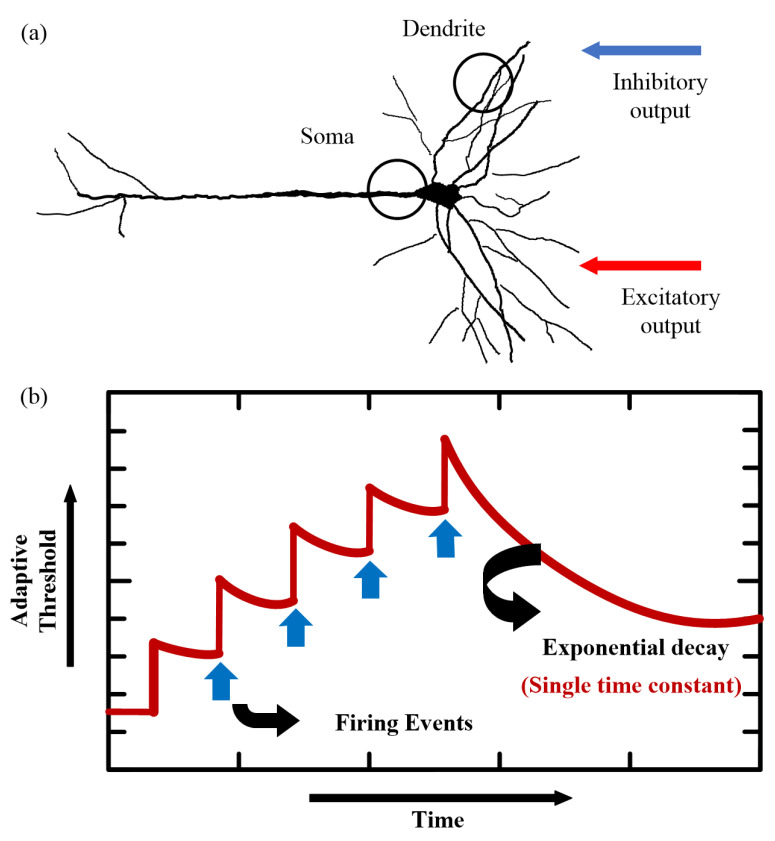
Dynamics of the proposed spiking neuron. (**a**) The biological structure that inspires the proposed neuron model. (**b**) The adaptive dynamics of the threshold along with the firing events.

Previous studies have shown that the firing timing and activity space of dendrites can significantly affect neural function. Excitability of dendrites can excite the membrane to fire, whereas inhibitory dendrites can have the opposite effect [26,27,28,29]. Inspired by this morphological structure and function of the neuron model, we propose a spiking neuron model, which has three compartments, including a somatic compartment and two dendritic compartments. The model utilizes distinct dendritic compartments to receive excitatory and inhibitory inputs, while using dendrites and somatic cells to receive and send spiking activities, respectively. The formulation for calculating the membrane potential of dendrites and soma are as follows

(1) $$\left\{ \begin{array}{l} \tau_{m}\frac{dU_{m}\left(t\right)}{dt}=-U_{m}\left(t\right)+R_{m}I_{m}\left(t\right)+g_{i}\left(U_{i}\left(t\right)-\theta_{i}\right)+g_{e}\left(U_{e}\left(t\right)-\theta_{e}\right)-\Gamma_{j}(t)z_{j}(t)\\ \tau_{i}\frac{dU_{i}\left(t\right)}{dt}=-U_{i}\left(t\right)+R_{i}I_{i}\left(t\right)\\ \tau_{e}\frac{dU_{e}\left(t\right)}{dt}=-U_{e}\left(t\right)+R_{e}I_{e}\left(t\right) \end{array}\right.$$

where *τ_v_* represents the time constant of membrane. The variables *U*(*t*), *U_i_*(*t*), and *U_e_*(*t*) represent the somatic membrane potentials, inhibitory dendritic membrane potentials, and excitatory dendritic membrane potentials, respectively. The parameters *θ_e_* and *θ_i_* represent the reversal membrane potential of excitatory dendrite and inhibitory dendrite, respectively. $R_{m}$, $R_{e}$, and $R_{i}$ represent the membrane resistance of the soma, excitatory dendrite, and inhibitory dendrite, respectively. The parameters *g_e_* and *g_i_* represent the synaptic conductance of excitatory dendrites and inhibitory dendrites, respectively. Neuron emits a spike at time *t* when it is currently not in a refractory period. The soma of neurons uses the spike adaptation mechanism. The threshold size can be changed by analyzing the firing pattern of neurons. Variable *z_j_*(*t*) represents the spike train of neuron *j* and assumes value in {0, 1/Δ*t*}. The dynamics of *Γ_j_*(*t*) is changed with each spike, representing the firing rate of neuron *j*, which is defined as

(2) $$\Gamma_{j}(t)=\tau_{j}^{0}+\alpha \cdot \tau_{j}(t)$$

where α represents a constant that scales the deviation *τ_j_*(*t*) from the baseline *τ_j_*^0^. The variable *τ_j_*(*t*) can be defined as

(3) $$\tau_{j}(t+\Delta t)=\beta_{j}\tau_{j}(t)+(1-\beta_{j})z_{j}(t)$$

where $\beta_{j}=\exp(-\Delta t/\tau_{a,j})$. The constant *τ_a,j_* represents the adaptation time constant. Variable *z_j_*(*t*) represents the spike train of neuron *j* and assumes value in {0, 1/Δ*t*}. The parameter values of the spiking neuron model that we proposed are listed in Table 1. The input current *I_j_*(*t*) of a neuron is defined as the weighted sum of the pulses, which come from external neurons or other neurons. Its mathematical formula is as follows

(4) $$\left\{ \begin{array}{l} I_{m}^{j}(t)=\sum_{j=1}^{n}W_{ij}^{}\chi_{i}^{}(t-\kappa_{ij}^{})+\sum_{j=1}^{n}W_{ij}^{rec}\varepsilon_{i}(t-\kappa_{ij}^{rec})\\ I_{i}^{j}(t)=\sum_{j=1}^{n}W_{ij}^{i}\chi_{i}^{}(t-\kappa_{ij}^{i})+\sum_{j=1}^{n}W_{ij}^{irec}\varepsilon_{i}(t-\kappa_{ij}^{irec})\\ I_{e}^{j}(t)=\sum_{j=1}^{n}W_{ij}^{e}\chi_{i}^{}(t-\kappa_{ij}^{e})+\sum_{j=1}^{n}W_{ij}^{erec}\varepsilon_{i}(t-\kappa_{ij}^{erec}) \end{array}\right.$$

where $W_{ij}^{rec}$, $W_{ij}^{erec}$, and $W_{ij}^{irec}$ represent the recurrent synaptic weights of soma, excitatory dendrites, and inhibitory dendrites, respectively. In addition,$W_{ij}^{}$, $W_{ij}^{e}$, and $W_{ij}^{i}$ represent the synaptic weights of soma, excitatory dendrite, and inhibitory dendrite, respectively. The constants $\kappa_{ij}^{}$, $\kappa_{ij}^{e}$, and $\kappa_{ij}^{i}$ represent the delays of input synapses for soma, excitatory dendrite, and inhibitory dendrite, respectively. The constants $\kappa_{ij}^{rec}$, $\kappa_{ij}^{erec}$, and $\kappa_{ij}^{irec}$ represent the delays of recurrent synapses for soma, excitatory dendrite, and inhibitory dendrite, respectively. The spike trains $\chi_{i}\left(t\right)$ and $\varepsilon_{i}\left(t\right)$ are modeled as sums of Dirac pulses, representing the spike trains from input neurons and recurrent neurons with recurrent connections, respectively. The dynamics of the proposed spiking neuron model are shown in Figure 1 accordingly.

**Figure 2:**
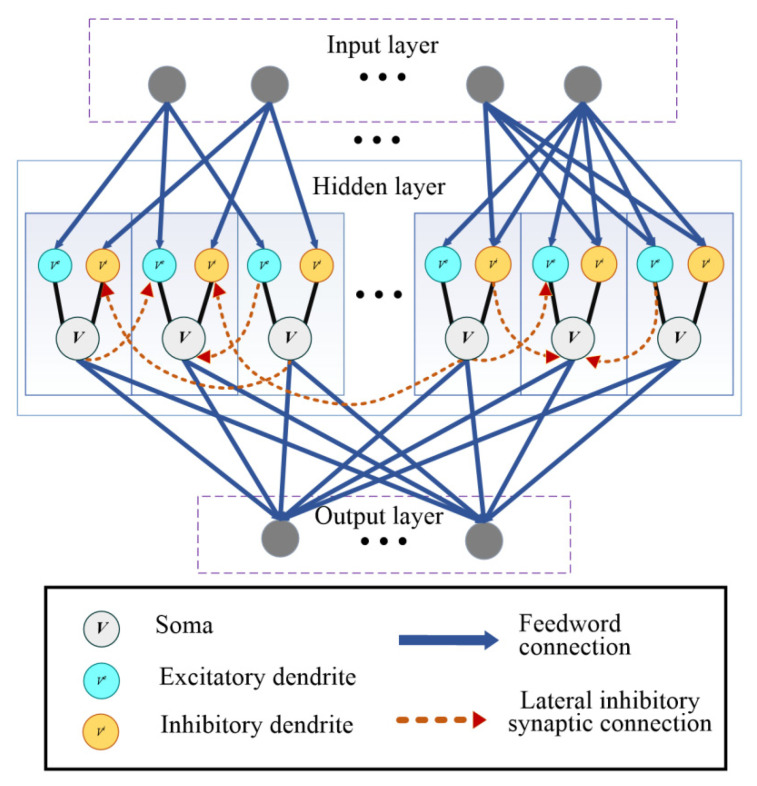
Network architecture for learning and memory integrated with the proposed SAM model. This network architecture is comparable to a 2-layer network of point neurons. The soma and dendrites of different neurons in the hidden layer are connected to lateral inhibitory synapses randomly. The gray circles in the input layer and output layer are not SAM neurons, representing the input spiking neuron and output spiking neuron, respectively. The input and output encodings are determined for different tasks, which will be described in the section of experimental results.

We integrate the spiking neuron model into an SNN framework and test the accuracy of this new model on different types of learning tasks. The structure of the SNN model is shown in Figure 2. The model is divided into three layers: input layer, hidden layer, and output layer. According to different tasks, we choose different encoding methods of the input layer and decoding methods of the output layer. In Figure 2, the solid blue lines represent feed-forward inhibitory synaptic connections, while the red dashed lines represent lateral inhibitory synaptic connections. The dendrites and soma of different neurons in the hidden layer are connected by lateral inhibitory synapses that are random and sparse at the same time. Information is transmitted from the input layer to the dendrites, and the soma transmits impulse signals to the output layer. The initial network weights in the proposed SNN model are set via a Gaussian distribution *W_ij_* ~ $\frac{w_{0}}{\sqrt{n_{in}}}N\left(0,1\right)$, where *n_in_* represents the number of input neurons in the spiking neural network in the weight matrix. *N*(0, 1) represents the Gaussian distribution with zero mean and unit variance, while *w*_0_ = Δ*t*/*R_m_* represents a weight-scaling factor depending on the time step Δ*t* and membrane resistance *R_m_*. This scaling factor is significant as it is used to initialize the spiking neural network with a practical firing rate needed for efficient training.

We use a deep rewiring algorithm because it is able to maintain the sign of each synapse during the learning process [30]. Hence, this sign is inherited from the initial weights of the network. In consideration of this, the model needs efficient and reasonable initialization weights for both excitatory and inhibitory neurons. To achieve this, we sample neurons from a Bernoulli distribution, generating the symbol sign *k_i_* ∈ {−1, 1} randomly. At the same time, to avoid the problem of exploding gradients, we scale the weights so that the largest eigenvalue is less than 1. A large square matrix is generated with the number of rows selected, ultimately with uniform probability. This square matrix is then multiplied by a binary mask, resulting in a sparse matrix, as a part of the depth rewiring algorithm that we mentioned before. This algorithm achieves the goal of maintaining the level of sparse connectivity in the network by dynamically disconnecting some synapses while reconnecting others. In this algorithm, we set the temperature parameter to 0 and the L1-norm regularization parameter to 0.01.

### 2.2. BPTT Training Algorithm

In common ANN models, the gradients of the loss function are obtained with respect to the weights in the network using back propagation. Nevertheless, the training method of back propagation cannot be directly applied to SNNs due to the non-differentiability of spikes from SNNs. Providing that time is discretized, the gradient needs to be propagated through continuous time or multiple time steps. To enable the SNN model to learn in the training process, we use a pseudo-derivative technique as shown below

(5) $$\frac{dz_{j}\left(t\right)}{dv_{j}\left(t\right)}=k\max\left\{0,1-\left|v_{j}\left(t\right)\right|\right\}$$

where *k* = 0.3 (typically less than 1) is a constant value that can dampen the increase in back propagated errors through spikes by using a pseudo-derivative of amplitude to achieve the goal of stable performance. The variable *z_j_*(*t*) represents the spike train of neuron *j* that assumes values in {0, 1}. The variable *v_j_*(*t*) represents the normalized membrane potential, which is defined as follows

(6) $$v_{j}\left(t\right)=\frac{V_{j}\left(t\right)-\Gamma_{j}\left(t\right)}{\Gamma_{j}\left(t\right)}$$

where *Γ_j_* represents the firing rate of neuron *j*. With the purpose of providing the self-learning capability required for reinforcement learning for the proposed SAM model, we utilize a proximal policy optimization algorithm [31]. This algorithm is easy to implement and allows the model to have self-learning capabilities. The clipped surrogate objective of this algorithm is defined as $O^{PPO}\left(\vartheta_{old},\vartheta ,t,k\right)$. Therefore, the loss function with respect to ϑ is formulated as

(7) $$L_{P}\left(\theta \right)=-\frac{\sum_{k<K}\sum_{t<T}O^{PPO}\left(\vartheta_{old},\vartheta ,t,k\right)}{KT}+\mu_{f}\frac{1}{n}{\sum_{j}\left\|\frac{\sum_{k,t}z_{j}\left(t,k\right)-f^{0}}{KT}\right\|}^{2}$$

where *f*^0^ represents a target firing rate of 10 Hz and *μ*_f_ represents a regularization hyperparameter. Variables *t* and *k* represent the simulation time step and the total number of epochs. The variable ϑ represents the current policy parameter, which is defined in the previous research [31]. In each iteration of training, *K* = 10 episodes of *T* = 2000 time steps are generated with a fixed parameter $\vartheta_{old}$, which is the vector of policy parameters before the update as expressed in [31]. At the same time, the loss function *L*(ϑ) is minimized by the ADAM optimizer [32].

### 2.3. Minimum Error Entropy Criterion (MEEC)

The minimum error entropy (MEE) can minimize the entropy of the estimation error, so that decreases the uncertainty in the learning process. The α-order Renyi’s entropy is used assuming a random variable *e* with probability density function *f^α^*(*e*), which is defined as

(8) $$H\left(e\right)≜\frac{1}{1-\alpha }\log\int f^{\alpha }\left(e\right)de$$

where α is set to 2 for 2-order Renyi’s entropy in this study. The kernel density estimation (KDE) is used to estimate the PDF of the error samples, which has three advantages. First, it is a non-parameter approach, which does not require the prior knowledge of the error distribution. Second, it does not require the integration calculation. Third, it can be smooth and differentiable, which is vital for the gradient computation. Considering a set of i.i.d data ${\left\{e_{i}\right\}}_{i=1}^{N}$ drawn from the distribution, the KDE of the PDF can be formulated as

(9) $${\hat{f}}_{E}\left(e\right)=\frac{1}{N}\sum_{i=1}^{N}G_{\sum }\left(e-e_{i}\right)$$

where *G_Σ_*(*e* − *e_i_*) represents the Gaussian function with the following expression as

(10) $$G_{\sum }\left(e-e_{i}\right)=\frac{1}{\sqrt{2\pi \left(\det\sum \right)}}\cdot \exp\left(-\frac{1}{2}{\left(e-e_{i}\right)}^{T}\sum^{-1}\left(e-e_{i}\right)\right)$$

where *N* and *Σ* represent the number of the data points and the kernel parameter, respectively. In this research, *Σ* represents a diagonal matrix with the *s*-th diagonal element with the variance $\delta_{s}^{2}$ for *e_s_* in *e*, where *s* = 1, 2, …, *S*. The kernel parameter represents a free parameter. Thus, the Renyi’s quadratic entropy can be expressed as

(11) $$\begin{array}{ll} H_{2}\left(e\right) & =-\log\int {\left(\frac{1}{N}\sum_{i=1}^{N}G_{\sum }\left(e-e_{i}\right)\right)}^{2}de\\ & =-\log\frac{1}{N^{2}}\int \left(\sum_{i=1}^{N}\sum_{j=1}^{N}G_{\sum }\left(e-e_{i}\right)G_{\sum }\left(e-e_{j}\right)\right)de^{}\\ & =-\log\frac{1}{N^{2}}\int \left(\sum_{i=1}^{N}\sum_{j=1}^{N}G_{\sum }\left(e-e_{i}\right)G_{\sum }\left(e-e_{j}\right)\right)de^{}\\ & =-\log\frac{1}{N^{2}}\left(\sum_{i=1}^{N}\sum_{j=1}^{N}G_{\sqrt{2}\sum }\left(e_{i}-e_{j}\right)\right)\\ & =-\log\frac{1}{N^{2}}\left(\sum_{i=1}^{N}\sum_{j=1}^{N}G_{\sum_{2}}\left(e_{i}-e_{j}\right)\right) \end{array}$$

Based on the Formula (11), we define a function *V*(*e*) to represent the information potential of variable *e*, which is formulated as

(12) $$V\left(e\right)=\frac{1}{N^{2}}\left(\sum_{i=1}^{N}\sum_{j=1}^{N}G_{\sum_{2}}\left(e_{i}-e_{j}\right)\right)$$

Therefore, the minimization of the Renyi’s entropy *H*_2_(*e*) means the maximization of the information potential *V*(*e*) because of the monotonic increasing feature of the log function. The Parzen window is used to decrease the computational complexity and the instantaneous information potential at time *t*, which can be formulated as

(13) $$J_{1}\left(e\right)=\frac{1}{W}\sum_{i=k-W+1}^{k}G_{\sum_{2}}\left(e_{k}-e_{i}\right)$$

where *W* represents the length of the Parzen window. It should be noted that MEE is a kind of local optimization criterion but suffers from the shift-invariant problem. It can only determine the location of error PDF but cannot know the distribution location. The function *G*_*Σ*2_(.) can be defined as the Gaussian kernel function with bandwidth *σ*

(14) $$G_{\sum 2}\left(x\right)=\frac{1}{\sqrt{2\pi }\sigma }\exp\left(-\frac{x^{2}}{2\sigma^{2}}\right)$$

In order to reduce the computational complexity, quantization technique is used to realize the quantized MEE (QMEE). Thus, the information potential is expressed as

(15) $$V^{Q}\left(e\right)=\frac{1}{N^{2}}\left(\sum_{i=1}^{N}\sum_{j=1}^{N}G_{\sum_{2}}\left(e_{i}-Q\left|e_{j}\right|\right)\right)=\frac{1}{N^{2}}\sum_{i=1}^{N}\sum_{j=1}^{M}\varphi_{j}G_{\sum 2}\left(e_{i}-c_{j}\right)$$

where *Q*[.] represents a quantization operator mapping each ${\left\{e_{i}\right\}}_{i=1}^{N}$ to one of ${\left\{c_{j}\right\}}_{j=1}^{M}$, resulting in a codebook *C* = (*c*_1_, *c*_2_, *c*_3_,…, *c*_M_). *Φ* = (*φ*_1_, *φ*_2_, …, *φ*_M_) represents the number of the samples quantized to the corresponding set ${\left\{c_{j}\right\}}_{j=1}^{M}$. It should be noted that $\sum_{j=1}^{M}\varphi_{j}=N$. Theoretical proof of the robustness has been presented in [22].

### 2.4. Restricted MEEC

In this study, the fundamental inner product to measure the similarity is used, which is generalized from its vectors’ application [33]. The inner product similarity between continuous pdfs *f_X_*(*x*) and *g_X_*(*x*) can be expressed as

(16) $$\langle f_{X}\left(x\right),g_{X}\left(x\right)\rangle =\int_{X}f_{X}\left(x\right)g_{X}\left(x\right)dx$$

The desired distribution *ρ_E_*(*e*), which is expressed in [33] in detail, can be defined as follows

(17) $$\rho_{E}\left(e\right)=\left\{ \begin{array}{l} \zeta_{0},\;\;\;\;e=0\\ \zeta_{-1},\;\;e=-1\\ \zeta_{1},\;\;\;\;e=1\\ 0,\;\;\;\;\;\mathrm{otherwise} \end{array}\right.$$

where *ζ_i_* (*i* = 0, −1, 1) denotes the corresponding density for each peak, which is simplified into a Dirac-δ function.

The maximization of the similarity measure between the error pdf *f_E_*(*e*) and the desired distribution *ρ_E_*(*e*) can be formulated as

(18) $$\begin{array}{l} \max\langle f_{E}\left(e\right),\rho_{E}\left(e\right)\rangle \\ \Leftrightarrow \max\int_{X}f_{E}\left(e\right)\rho_{E}\left(e\right)dx\\ \Leftrightarrow \max\zeta_{0}f_{E}\left(0\right)+\zeta_{-1}f_{E}\left(-1\right)+\zeta_{1}f_{E}\left(1\right) \end{array}$$

Furthermore, the model parameter can be expressed as

(19) $$\begin{array}{l} w^{*}=\arg\max\zeta_{0}{\hat{f}}_{E}\left(0\right)+\zeta_{-1}{\hat{f}}_{E}\left(-1\right)\zeta_{1}{\hat{f}}_{E}\left(1\right)\\ =\arg\max\left(\begin{array}{l} \zeta_{0}\frac{1}{N}\sum_{i=1}^{N}G_{\sum 2}\left(0-e_{i}\right)\\ +\zeta_{-1}\frac{1}{N}\sum_{i=1}^{N}G_{\sum 2}\left(-1-e_{i}\right)\\ \zeta_{1}\frac{1}{N}\sum_{i=1}^{N}G_{\sum 2}\left(1-e_{i}\right) \end{array}\right)\\ =\arg\max\frac{1}{N^{2}}\sum_{i=1}^{N}\left(\begin{array}{l} N\zeta_{0}G_{\sum 2}\left(e_{i}\right)\\ +N\zeta_{-1}G_{\sum 2}\left(e_{i}+1\right)\\ +N\zeta_{1}G_{\sum 2}\left(e_{i}-1\right) \end{array}\right) \end{array}$$

In fact, QMEE converges the prediction errors ${\left\{c_{j}\right\}}_{j=1}^{M}$ to obtain a compact error distribution. Based on the method in [33], a predetermined codebook *C* = (0, −1, 1) implements QMEE to restrict errors to three positions and avoid the undesirable double-peak learning consequence. Therefore, the restricted MEE (RMEE) algorithm can be formulated as

(20) $$V^{R}\left(e\right)=\frac{1}{N^{2}}\sum_{i=1}^{N}\left(\begin{array}{l} \varphi_{0}G_{\sum 2}\left(e_{i}\right)\\ +\varphi_{-1}G_{\sum 2}\left(e_{i}+1\right)\\ +\varphi_{1}G_{\sum 2}\left(e_{i}-1\right) \end{array}\right)$$

where *Φ* = (*φ*_0_, *φ*_−__1_, *φ*_1_) = (*Nζ*_0_, *Nζ*_−__1_, *Nζ*_1_) that represents the corresponding number for each quantization word *C* = (0, −1, 1). The proposed RMEE algorithm maximizes the inner product similarity between error pdf *f_E_*(*e*) and the optimal three-peak distribution *ρ_E_*(*e*). RMEE is a specific formation of QMEE where the codebook is predetermined as *C* = (0, −1, 1) and converges learning errors on these three locations.

In order to optimize Equation (19), the half-quadratic technique is used to solve optimization issues. A convex function *g*(*x*) = −*x*log(−*x*) + *x* is defined, and the information potential can be expressed as

(21) $$V^{R}\left(e\right)=\sum_{i=1}^{N}\left(\begin{array}{l} \varphi_{0}\left\{u_{i}\frac{e_{i}^{2}}{2\sigma^{2}}-g\left(u_{i}\right)\right\}\\ +\varphi_{-1}\left\{v_{i}\frac{{\left(e_{i}+1\right)}^{2}}{2\sigma^{2}}-g\left(v_{i}\right)\right\}\\ +\varphi_{1}\left\{s_{i}\frac{{\left(e_{i}-1\right)}^{2}}{2\sigma^{2}}-g\left(s_{i}\right)\right\} \end{array}\right)≜J_{R1}\left(w,u_{i},v_{i},s_{i}\right)$$

In half-quadratic technique, it has the following relationship

(22) $$\begin{array}{l} u_{i}^{k}=-\exp\left(-\frac{e_{i}^{2}}{2\sigma^{2}}\right)<0\\ v_{i}^{k}=-\exp\left(-\frac{{\left(e_{i}+1\right)}^{2}}{2\sigma^{2}}\right)<0\\ s_{i}^{k}=-\exp\left(-\frac{{\left(e_{i}-1\right)}^{2}}{2\sigma^{2}}\right)<0\\ \left(i=1,2,\ldots ,N\right). \end{array}$$

By attaining the optimal ($u_{i}^{k}$, $v_{i}^{k}$, $s_{i}^{k}$) in the *k*th iteration, the information potential can be formulated as

(23) $$V^{R}\left(e\right)=\sum_{i=1}^{N}\left(\begin{array}{l} \varphi_{0}u_{i}{\left(t_{i}-y_{i}\right)}^{2}\\ +\varphi_{-1}v_{i}{\left(t_{i}+1-y_{i}\right)}^{2}\\ +\varphi_{1}s_{i}{\left(t_{i}-1-y_{i}\right)}^{2} \end{array}\right)≜J_{R2}\left(w\right)$$

The *J_R_*_2_(*w*) can be optimized based on gradient-based methods because the objective function is differentiable and continuous. For example, the gradient of *J_R_*_2_(*w*) can be expressed as

(24) $$\frac{\partial }{\partial w}J_{R2}\left(w\right)=\sum_{i=1}^{N}\left(\begin{array}{l} \varphi_{0}u_{i}\frac{\partial {\left(t_{i}-y_{i}\right)}^{2}}{\partial w}\\ +\varphi_{-1}v_{i}\frac{\partial {\left(t_{i}+1-y_{i}\right)}^{2}}{\partial w}\\ +\varphi_{1}s_{i}\frac{\partial {\left(t_{i}-1-y_{i}\right)}^{2}}{\partial w} \end{array}\right)=-2\sum_{i=1}^{N}\left(\begin{array}{l} \varphi_{0}u_{i}e_{i}\\ +\varphi_{-1}v_{i}\left(e_{i}+1\right)\\ +\varphi_{-1}s_{i}\left(e_{i}-1\right) \end{array}\right)x_{i}y_{i}\left(1-y_{i}\right)$$

The detailed algorithm of the HQ-based optimization and its convergence analysis for RMEE are presented in [33].

## 3. Results

### 3.1. Proposed Network with RMEE Criterion

Since MEE has the shift-invariant feature, and estimation results based on MEEC will not always converge to the true value. A consideration is to combine the RMEE criterion with CEE for a global optimal solution. The cross-entropy loss function, also regarded as log loss, is the most commonly used loss function for back propagation. The cross-entropy loss function increases as the predicted probability deviates from the actual label, and can be described as follows

(25) $$L_{ce}\left({\hat{y}}_{i},y_{i}\right)=-\sum_{i}y_{i}\log\left({\hat{y}}_{i}\right)$$

In this paper, the label $l^{n}$ of each image is used, which is only assumed to be 1 for images belonging to the same class of images during testing, and 0 otherwise. The cross-entropy formula can be expressed as

(26) $$J_{2}=\sum_{n=1}^{5}-l^{n}\log\sigma \left(y^{20+20\cdot n}\right)-\left(1-l^{n}\right)\log\left(1-\sigma^{20+20\cdot n}\right)$$

where the output of the SNN model is only counted after all images are fully rendered. Therefore, for the novel criterion, the performance index can be formulated as

(27) $$J_{k}\left(e\right)=\mu \left[\sum_{i=1}^{N}\left(\begin{array}{l} \varphi_{0}u_{i}{\left(t_{i}-y_{i}\right)}^{2}\\ +\varphi_{-1}v_{i}{\left(t_{i}+1-y_{i}\right)}^{2}\\ +\varphi_{1}s_{i}{\left(t_{i}-1-y_{i}\right)}^{2} \end{array}\right)\right]+\left(1-\mu \right)\left[\sum_{n=1}^{5}\left(\begin{array}{l} -l^{n}\log\sigma \left(y^{20+20\cdot n}\right)\\ -\left(1-l^{n}\right)\log\left(1-\sigma^{20+20\cdot n}\right) \end{array}\right)\right]$$

where *μ* represents a weighting constant. In the supervised learning tasks, there only exist cross-entropy and RMEE, which is described in Equation (27).

### 3.2. Autonomous Navigation

We first apply the proposed SNN model in the agent navigation task, which requires the network to have reinforcement learning capabilities. The agent needs to learn to find objects in a 2D area and eventually be able to navigate to find objects at random locations in the area. This task is interrelated with the neuroscience paradigm of the well-known Morris water maze task, which is designed to study learning in the brain [34]. In this task, a virtual agent is simulated as a point in the 2D simulation arena and is controlled by the proposed SNN model. The position of the agent is configured randomly with a uniform probability in the overall arena at the beginning of an episode. The agent produces a small velocity vector of the Euclidean norm and selects an action at each time step. It receives a reward value ‘1’ after reaching the destination.

In the navigation task, the information *s*(*t*) of the current environment state and the reward score *r*(*t*) are received as input data by neurons in the input layer at each time step. The coordinate information of the position is encoded by the input neurons through the Gaussian population rate encoding method. Furthermore, each neuron in the input layer is assigned a coordinate value with a firing rate, which is defined as: *r*_max_ = exp(−100(*ξ_i_*-*ξ*)^2^), where *ξ_i_* and *ξ* represent the actual coordinate value and the preferred coordinate value, respectively. *r*_max_ is supposed to be set as 500 Hz. Moreover, the instantaneous reward *r*(*t*) is encoded by two sets of input neurons. In the first group, the neurons generate spikes in sync when a positive reward is received, while in the second group, the neurons generate spikes as long as the proposed SNN model receives a negative reward. The output of the network is represented by five readout neurons in the output layer with membrane potential *λ_i_*(*t*). The action vector *ζ*(*t*) = (*ζ_x_*(*t*), *ζ_y_*(*t*))*^T^* is used to determine the movement of the agent in the navigation task that we mentioned before. It is calculated from a Gaussian distribution with mean *μ_x_* = tanh(*λ*_1_(*t*)) and *μ_y_* = tanh(*λ*_2_(*t*)) as well as variances *Φ_x_* = *σ*(*λ*_3_(*t*)) and *Φ_y_* = *σ*(*λ*_4_(*t*)). In the end, the output of the last readout neuron λ_5_ is calculated to predict the value function *μ_θ_*(*t*). This predicts the expected discounted sum of future rewards *Ω*(*t*) = *Σ_t’_* > *t_γt’_ − t_ω_*_(*t’*)_, where *ω*(*t’*) represents the reward at time *t’* and *γ* represents the discount factor, whose value is usually 0.99. 

**Figure 3:**
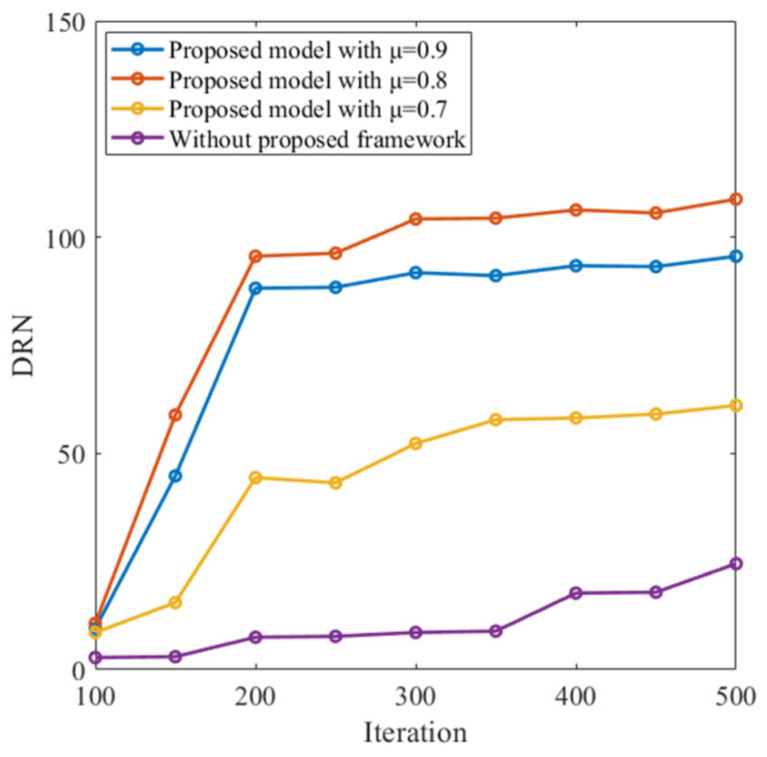
Navigation performance of the proposed model with different settings.

The agent based on the proposed SNN model learns to learn in the navigation task towards the correct destination location after the meta-learning process. The overall training process in the reward learning process is described by Algorithm 1. We add other loss functions to support the reinforcement learning framework, maintaining the loss function consistent with Equation (26). Figure 3 shows the successful destination reached number (DRN) per learning iteration. Each iteration contains a batch of ten episodes, and network weights are updated during the navigation task. For each episode, the model is expected to explore until reaching and storing the destination location, and uses the prior knowledge to find the shortest path to the destination. This reveals that the proposed SNN model has meta-learning capability in the autonomous navigation task.

**Algorithm 1** Training process in the reward learning process
**Input:** number of full episodes  K, timesteps T, fixed parameters $\theta_{old}$, target firing rate $f^{0}$, regularization hyper-parameters ${µ}_{v}$, ${µ}_{e}$, ${µ}_{firing}$, bandwidth σ, predicted value function $V_{\theta }\left(t,k\right)$ and sum of future rewards R(t,k) **Output:** total loss $L_{\theta }$.
    1.Parameters setting: $f^{0}$, ${µ}_{v}$, ${µ}_{e}$, ${µ}_{firing}\;and$ σ.    2.**for** n **in** batch size N:
    3.       Set $e_{n}=R\left(t,k\right)-V_{\theta }\left(t,k\right)$
    4.       **if** number of literation is 0:
    5.                 $\left(\varphi_{0},\;\;\varphi_{-1},\;\varphi_{1}\right)=\left(N,\;0,\;0\right)$
    6.       **else**:                
$\left(\varphi_{0},\;\;\varphi_{-1},\;\varphi_{1}\right)=(\#\left\{e_{n}\in \left(-0.5,\;0.5\right)\right\},$                                     
$\#\left\{e_{n}\in \left(-1,-0.5\right)\right\},$                                       
$\#\left\{e_{n}\in \left(0.5,\;1\right)\right\})$               
where #{·} indicates counting the samples that satisfy the condition
    7.        $\left(u_{n},v_{n},s_{n}\right)=\left(-exp\left(-\frac{e_{n}{}^{2}}{2\sigma^{2}}\right),-exp\left(-\frac{{\left(e_{n}+1\right)}^{2}}{2\sigma^{2}}\right),-exp\left(-\frac{{\left(e_{n}-1\right)}^{2}}{2\sigma^{2}}\right)\right)$
    8.        $L_{n}^{RMEE}$*=*$\varphi_{0}u_{n}e_{n}{}^{2}+\varphi_{-1}v_{n}{\left(e_{n}+1\right)}^{2}+\varphi_{1}s_{n}{\left(e_{n}-1\right)}^{2}$
    9. **end for**
   10. **for** k **in** K:
   11.        **for** t **in** T:
             $L_{\left(t,k\right)}^{PPO}=O^{PPO}\left(\theta_{old},\theta ,\;t,\;k\right)$   12.        **end for**
   13. **end for**
   14. Calculate the total loss:       $L\left(e\right)=L_{p}\left(e\right)+J_{k}\left(e\right)$
   15.**return** L(e)

### 3.3. Working Memory Performance on Store–Recall Task with Non-Gaussian Noise

**Figure 4:**
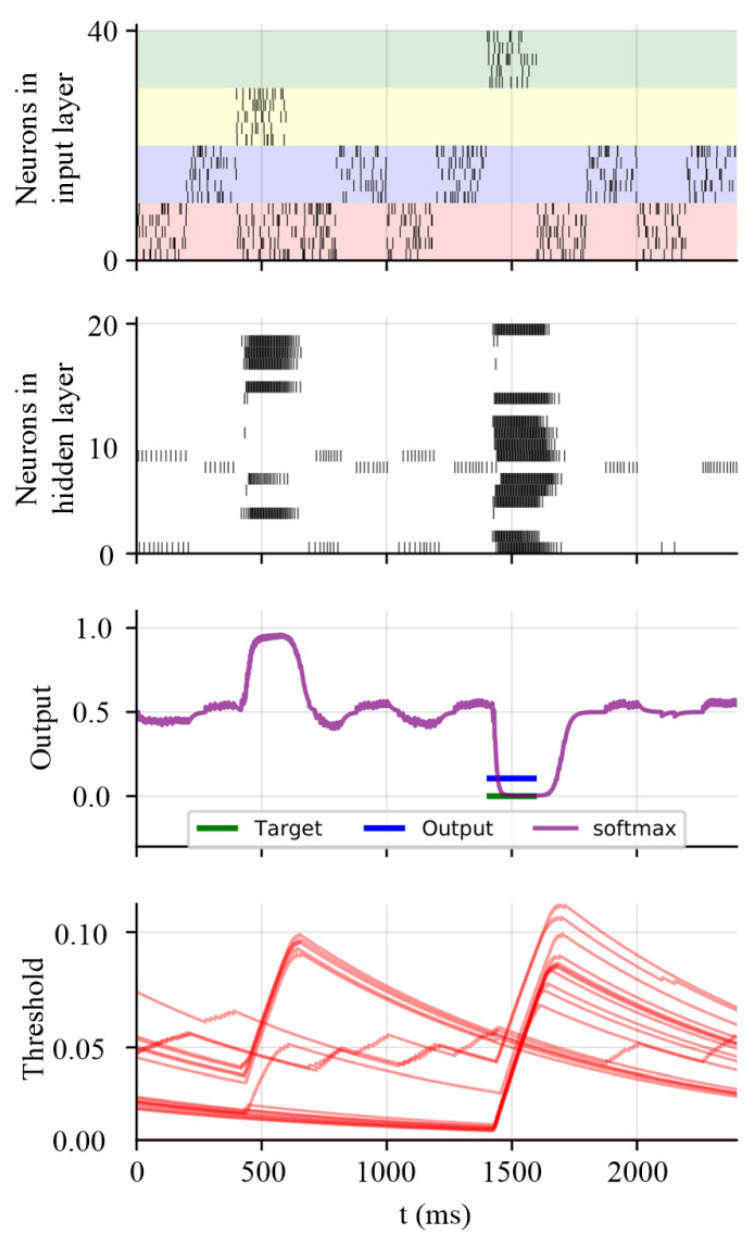
Working memory capability of the proposed SNN model after training.

To further demonstrate the robust working memory capability of the proposed SNN model, we apply the model in a store–recall task with non-Gaussian noise. The detailed settings of the store–recall task have been previously presented in [35]. The SNN model receives a sequence of frames that are represented by ten spike trains in a period of time. The inputs #1 and #2 are represented by the spiking activities of input neurons from #1 to #10 and from #11 to #20, respectively. As shown in Figure 4, the neurons from #21 to #30 and from #31 to #40 receive the random store and recall commands, respectively. The store command means direct attention is paid to the specific frame of input data flow. Then, this frame will be reproduced when receiving the recall command. Figure 4 shows one test example with the spiking activities after working memory training. The dynamic threshold changes along with the learning procedure, which is shown in Figure 4. This reveals that the proposed SNN model can exhibit the working memory performance and realize the store–recall task successfully. Since working memory is a vital feature and the foundation for meta-learning, this also suggests that the MeMEE model can exhibit the meta-learning tasks based on its working memory mechanisms with a robust performance.

### 3.4. Meta-Learning Performance on Sequential MNIST Data Set with Non-Gaussian Noise

**Figure 5:**
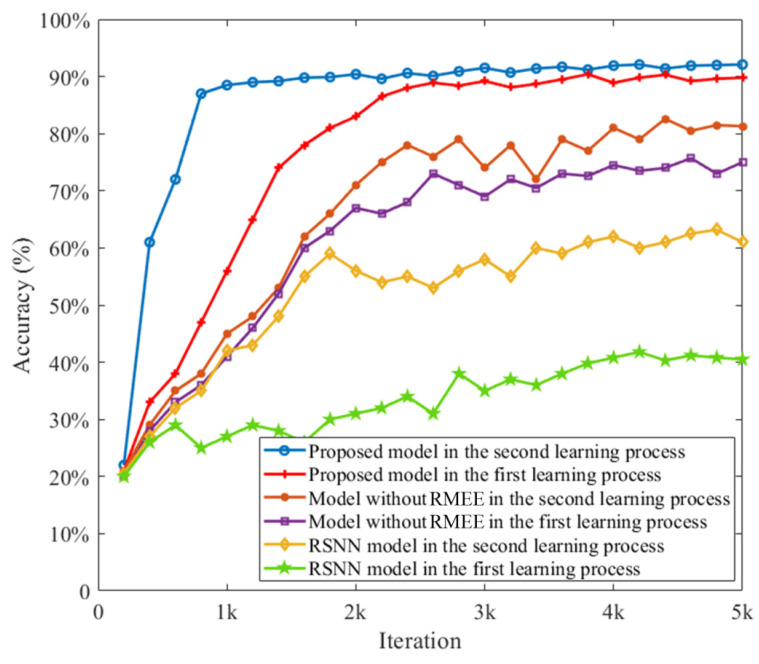
Meta-learning capability of the proposed MeMEE model on sequential MNIST data set.

We further demonstrate the meta-learning capability of the proposed SNN model in a transfer learning task based on the sequential MNIST (sMNIST) data set. We divide the sMNIST data set into two parts. The first part includes 30,000 images for digits ‘0’, ‘1’, ’2’, ‘3’, and ‘4’, and the second part includes 30,000 patterns for digits ‘5’, ’6’, ‘7’, ‘8’, and ‘9’. In the first phase, the first part is employed to train the SNN model, and the second part is then used for training. In the second phase, 10% salt and pepper noise is added to the testing data set as the non-Gaussian noise for the performance evaluation. Figure 5 shows the performance of the MeMEE model and compares it with other counterpart models, including recurrent SNN (RSNN) and the conventional LIF-based SNN model without the RMEE criterion. This shows that the proposed model outperforms the other solutions, and the reasoning behind this includes three points. Firstly, the proposed model has the meta-learning capability, so it can illustrate the transfer learning capability, and its transfer learning performance is superior to the RSNN model accordingly, considering accuracy and convergence speed. Secondly, due to the RMEE criterion being the loss function, its robustness to the non-Gaussian noise is superior to the model without the RMEE criterion in terms of the learning accuracy. The result suggests that the MeMEE model with RMEE criterion has a more powerful robust meta-learning capability in learning sequential spatio-temporal patterns.

### 3.5. Effects of Loss Parameters on Learning Performance

**Figure 6:**
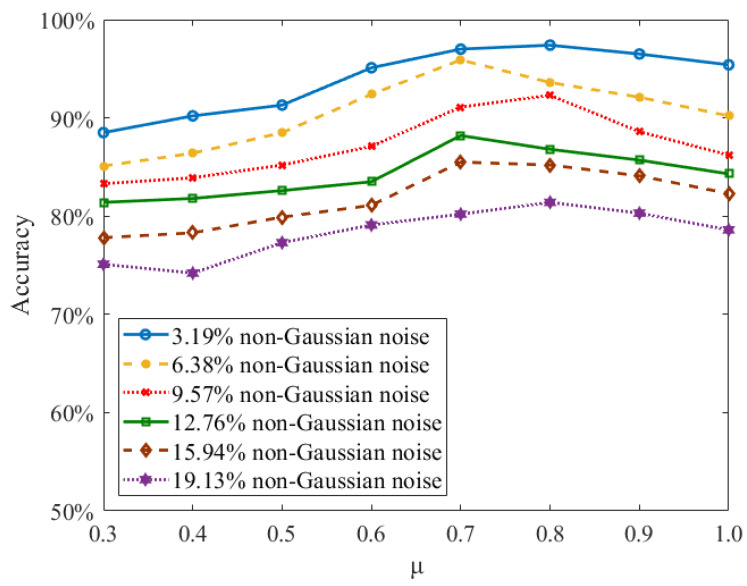
Effects of loss parameters on the learning performance of sequential classification.

In this study, we further investigate how each loss function affects the learning performance of the proposed MeMEE model. We use the sMNIST data set to evaluate and quantify the learning accuracy along with the changing loss parameter. In order to demonstrate the learning robustness based on the proposed MeMEE model, salt and pepper noise is added to the sMNIST data set. Different levels are considered, which are selected from 3.19% to 19.13%. Different values of parameter *μ* are investigated, which are set from 0.3 to 1.0. As shown in Figure 6, the value of *μ* with 0.7, 0.8, and 0.9 can induce the higher learning accuracy on sequential visual recognition. This reveals that the RMEE criterion can further enhance the robustness of the proposed MeMEE model without the RMEE criterion, i.e., *μ* = 1. Since the model without RMEE criterion with 3.19% non-Gaussian noise only reaches 83.6% accuracy, the RMEE criterion can improve the learning accuracy of the proposed MeMEE model with non-Gaussian salt and pepper noise.

## 4. Discussion

This paper presents an information theoretic learning framework for robust spike-driven continual meta-learning. Different from the previous SNN learning research, we first introduce the RMEE criterion to develop and improve the spike-based learning framework, which is significantly general and can also provide a series of theoretic insights. Moreover, the information theoretic framework allows us to obtain a direct understanding and better interpretation of the robust learning solutions of SNN models, compared with some previous studies focusing on improving the learning robustness of SNNs [36]. 

As a first step in establishing a rigorous framework for SNN continual meta-learning with RMEE, the presented research can be extended in both theoretical and practical aspects. From the theoretical point of view, one extension is to use the information potential to train the presented SNN model. For example, as shown in [37], Chen et al. presented a survival information potential algorithm for adaptive system training. This does not require computing of the kernel function and has good robustness performance accordingly. The other extension is to apply the proposed framework in other spike-based learning paradigms, including few-shot learning, multitask learning, and unsupervised learning [38].

From a practical point of view, the model is expected to be implemented on neuromorphic platforms to realize low-power and real-time systems for various types of applications. The state-of-the-art digital neuromorphic systems include Loihi [12], Tianjic [11], BiCoSS [13], CerebelluMorphic [14], LaCSNN [15], TrueNorth [39], and SpiNNaker [40]. By implementing embedded neuromorphic systems, it can be applied in different fields such as edge computing devices, brain–machine integration systems, and intelligent systems [41,42,43].

## 5. Conclusions

In this invited paper, we first presented an ITL-based scheme for robust spike-based continual meta-learning, which is improved by the RMEE criterion. A gradient descent learning principle is presented in a recurrent SNN architecture. Several tasks are realized to demonstrate the learning performance of the proposed MeMEE model, including autonomous navigation, robust working memory in the store–recall task and robust meta-learning capability for the sMNIST data set. In the first autonomous navigation task, the SNN model learns to find the correct destination by continual meta-learning from the task reward and punishment. This demonstrates that the MeMEE model based on the proposed RMEE criterion realizes the meta-learning capability for navigation and outperforms the conventional RSNN model. In the second task, the proposed MeMEE model improves the working memory performance by recalling the stored noisy patterns. In the third task, the proposed MeMEE model with RMEE criterion can enhance the robustness in the meta-learning task for noisy sMNIST images. This invited paper provides a novel insight into the improvement of the spike-based machine learning performance based on information theoretic learning strategy, which is critical for the further research of artificial general intelligence. In addition, it can be implemented by the low-power neuromorphic system, which can be applied in edge computing of internet of things (IoT) and unmanned systems. 

## Figures and Tables

**Table 1:** Parameter settings of the spiking neuron model.

Parameter	Value	Parameter	Value
*R_m_*	1 Ω	*R_i_*, *R_e_*	1 Ω
*τ_m_*	20 ms	*θ_i_*, *θ_e_*	0 mV
*κ*, *κ^i^*, *κ^e^*	5 ms	*κ^rec^*, *κ^irec^*, *κ^erec^*	5 ms
*α*	1.8	τ^0^	0.01
*τ_a_*	700 ms	*g_i_*, *g_e_*	1 nS
